# Publishing in the *Journal of Public Health in Africa*: Advancing research for future pandemics

**DOI:** 10.4102/jphia.v15i1.869

**Published:** 2024-11-27

**Authors:** Kumba Seddu, Nicaise Ndembi

**Affiliations:** 1 Johns Hopkins Bloomberg School of Public Health, Baltimore, Maryland, United States of America; 2 Harvard Medical School, Boston, Massachusetts, United States of America; 3 Africa Centres for Disease Control and Prevention, Addis Ababa, Ethiopia; 4 University of Maryland School of Medicine, Baltimore, Maryland, United States of America

As the world continues to recover from the COVID-19 pandemic, it has become evidently clear that future pandemic preparedness depends on global collaboration. Africa’s public health landscape is shaped by both regional uniqueness and global relevance. The *Journal of Public Health in Africa* (JPHIA), owned by the Africa Centres for Disease Control and Prevention (Africa CDC), offers a vital platform for fostering cross-continental research publications and collaborations that are essential for anticipating and preventing the next global health crisis.^1^

The publishing landscape in Africa has undergone notable changes, marked by an increase in output, particularly from countries like South Africa, Nigeria and Senegal.^2^ According to the *Global Research Report – Africa*, while the volume of research is growing, much of it is often published in international journals that do not adequately reflect or address the continent’s specific needs and challenges.^2^ This trend highlights a critical gap in the accessibility of research relevant to African contexts. The Rio de Janeiro Declaration on Health Sovereignty, established during the Global Pandemic Preparedness Summit 2024, emphasises the need for countries in the Global South to enhance their capabilities in developing research to address public health emergencies of international concern (PHEIC).^3^ Many African scholars face barriers to publishing both locally and globally, underscoring the importance of establishing and promoting platforms that prioritise African research and perspectives in the global research community.^2^

The Africa CDC created a platform to advance research through its own JPHIA. By publishing in JPHIA, researchers can ensure their work reaches a targeted audience invested in African public health challenges, fostering regional and global collaboration and enhancing the impact of their findings on policy and practice. Furthermore, by publishing in JPHIA, researchers contribute to an international knowledge base that helps safeguard global health security while strengthening local capacities and homebrew policy formulations by public health agencies in Africa, a region at the frontlines of emerging and re-emerging infectious diseases.^4^

## What is the *Journal of Public Health in Africa*?

The JPHIA is a peer-reviewed, open-access, online-only academic journal dedicated to addressing health challenges across the African continent. Its mission is to bring public health to the forefront of Africa’s fight against diseases, emphasising the critical role of public health strategies in shaping the continent’s health outcomes and advancing long-term solutions.^1,5^

## Why publish in the *Journal of Public Health in Africa*?

Publishing in the *Journal of Public Health in Africa* offers a unique opportunity to address global health challenges, advance cross-continental research collaboration and equity, and strengthen health systems while supporting the next generation of research scholars.

### Africa’s health challenges are global challenges

Africa’s public health issues – ranging from infectious diseases such as malaria, tuberculosis, and human immunodeficiency virus (HIV) or acquired immunodeficiency syndrome (AIDS), to emerging threats like non-communicable diseases (NCDs) and climate-sensitive health risks – do not remain confined to the continent; they have global repercussions.^6,7^ These challenges intersect with global public health, given the interconnectedness of populations, economies and climate change. Risk assessment of pandemics is increasingly critical as climate change exacerbates factors that contribute to the emergence and spread of infectious diseases. As global temperatures rise, the geographical distribution of pathogens and vectors, such as mosquitoes and ticks, may expand, facilitating the spread of diseases that were previously limited to specific regions.^8^ This intersection of climate change and pandemic risk underscores the need for integrated approaches to health and environmental policies, emphasising the importance of monitoring and mitigating risks to enhance resilience against future health crises. By publishing in the JPHIA, researchers from around the world contribute to addressing these shared health threats, ensuring information dissemination, which is crucial for rapidly evolving fields like public health and precision medicine, where timely access to research findings can influence policy and best practices. By serving as a platform for researchers worldwide, JPHIA enables the dissemination of crucial health findings and innovative solutions that address these intersecting global challenges.

### Promotes research collaboration and equity

A significant aspect of JPHIA’s mission is its promotion of research collaboration between African researchers and their global counterparts. Because public health issues transcend geographic borders, JPHIA serves as a unique platform that is not only mutually beneficial but essential for advancing solutions to health issues. These collaborations and co-authorships enrich the global scientific community by contributing fresh perspectives that enhance the understanding of diseases, health systems and the socio-economic factors that influence health outcomes and information that are essential for tackling global health challenges. In addition, equitable research collaboration enhances the relevance and applicability of health interventions. Solutions developed through inclusive, collaborative research efforts are more likely to be effective in diverse settings. This type of collaboration also strengthens the global response to emerging and re-emerging diseases. Epidemics like COVID-19, dengue, Ebola virus disease, Marburg virus disease, mpox and other zoonotic diseases highlight the need for a coordinated, global approach to disease surveillance, preparedness and response.

### Strengthening health system capacity and empowering the next generation of public health leaders

Africa is home to a growing population of young vibrant researchers eager to address the continent’s pressing health challenges while also contributing to global health solutions. They are leveraging artificial intelligence (AI) and digital tools to address pressing challenges, including public health issues. The World Bank and African Development Bank estimate that there are about 650 million mobile users in Africa, a number greater that its counterparts in Europe and United States (US).^9^ Artificial intelligence has the potential to revolutionise public health by improving diagnostics, predicting outbreaks and enhancing access to care, particularly in underserved regions. Despite Africa’s AI readiness scores – 38.59 for North Africa and 29.38 for sub-Saharan Africa, both below the global average – youth-led innovations are bridging these gaps and are positioning the continent at the forefront of the Fourth Industrial Revolution (4IR) technologies, specifically leading the way for AI in healthcare.^10^ The *Journal of Public Health in Africa* is a platform for early-career African researchers to publish their work and build a reputation in the research community. It helps ensure that their work is recognised and integrated into the global discourse on public health and disseminated to 55 African Union Member States. As these young researchers rise in prominence, they contribute to a stronger, more self-reliant African scholastic community that is capable of addressing the continent’s unique health challenges while contributing to international efforts to improve global health outcomes.

### An opportunity for African diaspora to contribute to the African renaissance dream

According to African Union’s Citizens and Diaspora Directorate (CIDO), there are 19m African diaspora globally.^11^ During health crises, including epidemics and pandemics, the African diaspora continues to play a critical role in assisting to end these crises through advocacy and fundraising, sending remittances and leading innovative initiatives.^11^ Through JPHIA, African diaspora researchers, scientists and public health professionals hold tremendous potential to advance the Africa Renaissance. With vast knowledge, skills and experiences gained globally, members of the diaspora can bring innovative solutions to address health challenges in Africa. Their involvement can enhance local research, strengthen health systems, and promote the exchange of best practices across continents. Publishing in the Africa CDC’s JPHIA enables researchers to not only push the boundaries of public health knowledge but also actively shape Africa’s journey towards self-reliance and sustainable health progress.^11,12^
